# Evidence that synergism between potassium and nitrate enhances the alleviation of ammonium toxicity in rice seedling roots

**DOI:** 10.1371/journal.pone.0248796

**Published:** 2021-09-09

**Authors:** Gen Fang, Jing Yang, Tong Sun, Xiaoxin Wang, Yangsheng Li

**Affiliations:** State Key Laboratory of Hybrid Rice, College of Life Sciences, Wuhan University, Wuhan, China; University of Massachusetts Amherst, UNITED STATES

## Abstract

Ammonium toxicity in plants is considered a global phenomenon, but the primary mechanisms remain poorly characterized. Here, we show that although the addition of potassium or nitrate partially alleviated the inhibition of rice seedling root growth caused by ammonium toxicity, the combination of potassium and nitrate clearly improved the alleviation, probably via some synergistic mechanisms. The combined treatment with potassium and nitrate led to significantly improved alleviation effects on root biomass, root length, and embryonic crown root number. The aberrant cell morphology and the rhizosphere acidification level caused by ammonium toxicity, recovered only by the combined treatment. RNA sequencing analysis and weighted gene correlation network analysis (WGCNA) revealed that the transcriptional response generated from the combined treatment involved cellulose synthesis, auxin, and gibberellin metabolism. Our results point out that potassium and nitrate combined treatment effectively promotes cell wall formation in rice, and thus, effectively alleviates ammonium toxicity.

## Introduction

Compared with nitrate, ammonium is the preferred nitrogen source for paddy rice and many other plant species. While a low supply of ammonium promotes plant growth, a high supply of ammonium causes toxicity, especially when applied as an exclusive nitrogen source [1]. At low concentrations (< 3 mM), ammonium is typically the nitrogen source preferred by plants, but above a certain threshold, ammonium becomes toxic [2]. Ammonium toxicity can lead to plant growth retardation, biomass reduction, structural changes, the formation of short and dense root systems, and even death [3]. In addition, ammonium toxicity inhibits root cell elongation and affects root gravitropism by disturbing auxin distribution in root tips [4–6].

Cells that absorb excess ammonium actively excrete ammonium. These transport processes utilize many ion channels, resulting in a large waste of energy [7]. To maintain the charge balance, plant cells need to pump out protons to counteract the excess ammonium in the cytoplasm, which causes rhizosphere acidification [8]. Ammonium can enter cells through a variety of nonselective cation channels and potassium-specific channels; transport through the latter competitively inhibits uptake of potassium [9, 10]. Excessive ammonium assimilation in the cytoplasm consumes a large amount of α-ketoglutaric acid, which decreases the amount of carbon available for metabolism [11]. The auxin signal in *Arabidopsis thaliana* roots is obviously reduced under ammonium treatment but exogenous application of auxin is unable to completely alleviate the toxicity phenotype in roots [12]. Ammonium toxicity can result in higher lipid peroxidation levels, a higher ratio of the oxidized state of glutathione and ascorbic acid, and higher activity of ROS-scavenging enzymes [13].

Plant ammonium toxicity can be alleviated, for instance, by increasing the activity of the ammonium assimilation system, relieving rhizosphere acidification, inhibiting futile transmembrane ammonium cycling, and increasing the supply of other nutrients [1–3]. Potassium has a significant alleviation effect on ammonium toxicity [14]. A possible reason for this effect is that potassium can competitively suppress the transport of ammonium, thereby reducing futile transmembrane ammonium cycling [15]. Some researchers have reported that the presence of nitrate in the growth medium ameliorates the ammonium toxicity symptoms, even at a very low nitrate concentration [9]. Since nitrate is an essential signaling molecule, the alleviation effect of nitrate on ammonium toxicity could be complex [16]. Nitrate, as an anionic and oxidized nitrogen source, can mitigate the imbalance of ions and redox states caused by cations and reduce ammonium ions when applied together [17]. The rhizosphere becomes alkalized when plants absorb nitrate, which counteracts the acidification caused by ammonium absorption [2]. Nitrate alleviates ammonium toxicity without lessening ammonium accumulation, organic acid depletion, and inorganic cation depletion in *A*. *thaliana* shoots [18].

In this study, we compared the alleviation effects of potassium and nitrate when given alone (as KCl and NaNO_3_) or combined (as KNO_3_) on ammonium toxicity and found that the combined application of potassium and nitrate not only improved the alleviation of ammonium toxicity compared with the application of individual ions but also resulted in some novel beneficial mechanisms, probably due to an undiscovered synergism. We provide morphological and physiological evidence of the existence of this synergism, and we performed a transcriptome analysis to reveal a regulatory network in cell wall construction related to this synergism.

## Materials and methods

### Plant materials and growth conditions

The plant materials used in this research included ZH11 (*Oryza sativa japonica*) and DR5:GUS insertion lines (ZH11 background) [19].

Rice seeds were germinated in distilled water for 2 days in a dark environment at 30°C and then transferred into hydroponic culture media. The growth density of the seedlings was strictly controlled at 24 plants per 400 mL medium and grown in a growth chamber (30°C-14 hour-light/22°C-8 hour-dark cycles, 100 μmol m^−2^ s^−1^ illumination and 80% humidity).

There were 6 groups of hydroponic culture media used in this research: control (pure water); ammonium toxicity treatment (4 mM NH_4_Cl solution); potassium alleviation treatment (4 mM NH_4_Cl and 4 mM KCl solution); nitrate alleviation treatment (4 mM NH_4_Cl and 4 mM NaNO_3_ solution); potassium and nitrate alleviation treatment (4 mM NH_4_Cl and 4 mM KNO_3_ solution); sodium and chlorine alleviation treatment (4 mM NH_4_Cl and 4 mM NaCl solution). No other nutrients were added to the hydroponic culture media except for the above mentioned ions.

If not mentioned, 7-day-old seedlings were harvested for experiments.

### Analysis of root morphology

Seedlings were removed from the boxes, and the roots were immersed in water contained in petri dishes. The petri dishes were placed on a black cloth, and the whole root area per plant was photographed using a D7000 camera (Nikon, Japan). All the root morphology parameters were measured with SmartRoot software V4.2 [20] according to the user guide.

The root tips were cut and fixed with FAA fixative (1.9% formaldehyde, 4.9% acetic acid, 63% ethanol, wt/vol) for 2 days and then embedded in paraffin. Then, 6-μm-thick slices were obtained with a microtome. After dewaxing with xylene, the sections were placed on glass slides and photographed using a microscope (DM4000B equipped with a DFC490 camera, Leica, Germany).

### Culture pH measurement

At selected times after germination, the pH of the hydroponic culture media was measured using a pH meter (PB-10, Sartorius, Germany).

### DR5:GUS-based auxin assay

The DR5:GUS insertion line grown as above was harvested and rinsed in distilled water for 30 s. After fixing in 90% (wt/wt) acetone in vacuo, the roots were rinsed with GUS staining buffer (50 mM Na_2_HPO_4_, 50 mM NaH_2_PO_4_, 0.1% Triton X-100, 2 mM K_4_[Fe(CN)_6_], 10 mM EDTA) 3 times and then stained with 5 mL of GUS staining buffer containing 5 μg of X-gluc (Sigma-Aldrich, USA) for 2h, then they were immersed in the GUS staining buffer for observation. Images were photographed under a microscope (DM4000B equipped with a DFC490 camera, Leica, Germany).

### Ion content, enzyme activity, and soluble protein content determination in roots

Seedling roots were harvested and rinsed in distilled water for 30 s to remove extracellular ions. About 2 g roots from each treatment were homogenized (1 min for each sample) under liquid N_2_ using a mortar and pestle.

For tissue ammonium content determination, 0.2 g of homogenized tissue was mixed with 2 mL of 10 mM precooled formic acid before centrifugation (4°C, 10000 ×g, 10 minutes). The supernatants were analyzed by the o-phthalaldehyde (OPA) method to determine the ammonium content using spectrophotometry as described in [21].

For tissue nitrate content determination, 0.1 g of homogenized tissue was mixed with 1 mL of H_2_O before centrifugation (4°C, 10000 ×g, 10 minutes). Then, 30 μL of supernatant was mixed with 100 μL of 5% (wt/vol) salicylic acid-H_2_SO_4_. After reacting for 20 minutes at room temperature, 2 mL of 2 M NaOH was added to the mixture. The sample absorbance was measured at 410 nm, and the nitrate content was computed according to a standard curve.

The potassium content was determined by the atomic absorption spectroscopy (AAS) method using a contraAA700 (Analytik Jena, Thuringia, Germany). After determining the dry root weights, the samples were digested with 5 mL of concentrated nitric acid and 1 mL of H_2_O_2_ at 150°C until the mixtures were transparent. The solutions were then diluted to 50 mL with 0.5% nitric acid. The potassium content was determined at 404.4 nm.

Homogenized tissue was lysed with Western and IP lysis buffer (Beyotime Biotechnology, Shanghai, China). The soluble protein content was measured with a BCA protein assay kit (Beyotime Biotechnology, Shanghai, China).

Glutamine synthetase (GS) activity was measured using a GS activity assay kit (Comin Biotechnology, Suzhou, China) as described in [22].

Glutamate synthase (GOGAT) activity was measured using a GOGAT activity assay kit (Comin Biotechnology, Suzhou, China) as described in [23].

### Net ^15^NH_4_ flux measurement

Rice seedlings were germinated and grown on 4 mM ^14^NH_4_Cl for 7 days. After harvesting, their roots were rinsed with 0.1 mM CaSO_4_ immediately for 1 minute. Then, the samples were evenly separated into 4 groups and immersed in 4 mM ^15^NH_4_Cl, 4 mM ^15^NH_4_Cl + 4 mM KCl, 4 mM ^15^NH_4_Cl + 4 mM Na^14^NO_3_, or 4 mM ^15^NH_4_Cl + 4 mM K^14^NO_3_ for 1 hour. After rinsing again with 0.1 mM CaSO_4_ for 1 minute, the roots from the 4 groups were dried in a 70°C oven for 48 hours. Then, they were homogenized using liquid N_2_. The proportions of ^15^N in the total nitrogen content (wt/wt) in the homogenized roots, which represents the root surface ^15^NH_4_ influx in 1 hour, were measured by isotope mass spectrometry (isoprime-100, Vario ISOTOPE cube CN).

The method for root surface ^15^NH_4_^+^ efflux (in 1 hour) measurement is the same as above, except for the following part. Rice seedlings were germinated and grown on 4 mM ^15^NH_4_Cl solution for 7 days, and they were immersed in 4 mM ^14^NH_4_Cl, 4 mM ^14^NH_4_Cl + 4 mM KCl, 4 mM ^14^NH_4_Cl + 4 mM Na^14^NO_3_, or 4 mM ^14^NH_4_Cl + 4 mM K^14^NO_3_ for 1 hour. Finally, the proportions of ^15^N in the total nitrogen content (wt/wt) in the homogenized roots were measured, which represents the root surface ^15^NH_4_^+^ efflux in 1 hour.

^15^NH_4_Cl was obtained from Shanghai Engineering Research Center of Stable Isotope (Shanghai, China), and 99% of the nitrogen atoms were ^15^N.

### RNA sequencing and data analysis

Total RNA was extracted from 15 samples of ZH11 roots under 5 different treatments (3 biological replications for each treatment, 40 plants for each biological replication). The cDNA libraries were constructed by Personal Biotechnology (Shanghai, China) using the TruSeq RNA Sample Prep Kit (Illumina, USA). The mRNA paired-end sequencing was performed using the NextSeq 500 platform (Illumina, USA) by the same company. The raw RNA sequencing reads have been deposited in the NCBI Sequence Read Archive (SRA) under accession No. PRJNA693667.

After removing the adapter sequences from raw reads, clean reads were obtained by Trimmomatic [24]. The clean reads were mapped to the reference genome of the *Oryza sativa japonica* variety (ftp://ftp.ensemblgenomes.org/pub/plants/release-36/fasta/oryza_sativa/dna/Oryza_sativa.IRGSP-1.0.dna.toplevel.fa.gz) using STAR [25], and gene structure information was obtained from EnsemblPlants annotation (ftp://ftp.ensemblgenomes.org/pub/plants/release-36/gtf/oryza_sativa/Oryza_sativa.IRGSP-1.0.36.chr.gtf.gz). The normalized gene expression levels (TMM normalized FPKM) were calculated using Trinity [26]. DESeq2 [27] was used to identify the differentially expressed genes. Those with an adjusted p-value (padj) < 0.05 and |log_2_FC| > 1 genes between every pair of groups were considered significantly differentially expressed genes.

### Weighted gene correlation network analysis (WGCNA)

A matrix containing the TMM values of all genes was used as an input file, and 40% of the genes were first filtered out using the varFilter command (var.func = IQR) in the R package genefilter. Then, WGCNA was performed using the R package WGCNA [28]. A suitable beta value of 18 was calculated, and the TOMType was selected as "signed" to construct a coexpression matrix, which divided genes into 15 modules, including 14 valid modules and 1 “gray” module. The correlations between the modules and traits were calculated to select the modules for further analysis. Hub genes were selected using a module membership > 0.9 and a gene significance > 0.8.

## Results

### Growth, morphology, and auxin distribution

**Fig 1:**
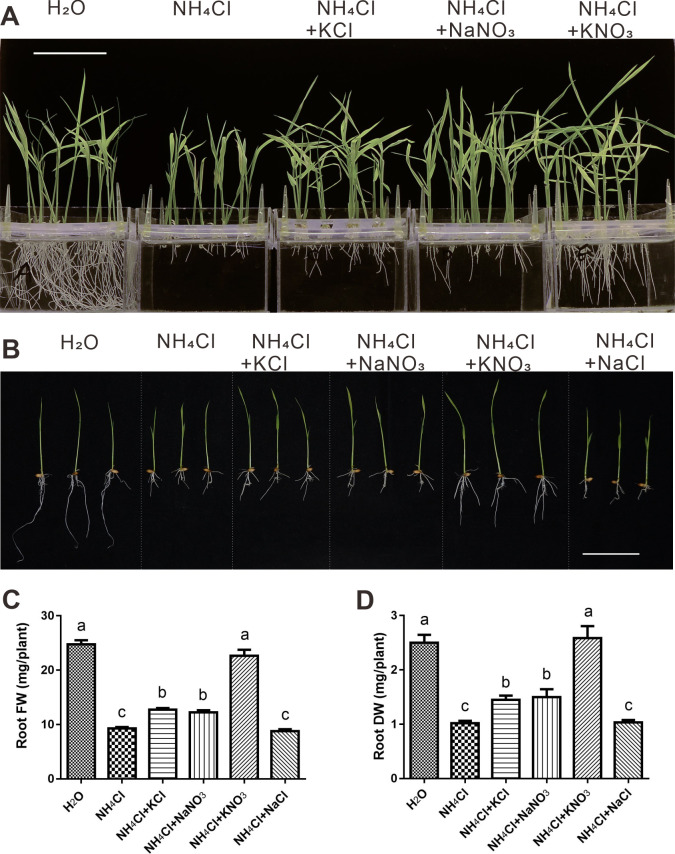
Phenotypes of rice seedling roots under. (A) Photograph of 10-day-old rice seedlings. (B) Photograph of 7-day-old rice seedlings. (C and D) Fresh weight (FW) and dry weight (DW) of roots of 7-day-old seedlings. Scale bars = 5 cm. Data are mean ± SD; t-tests were used to identify significant differences, and different letters represent significant differences among different treatments (p < 0.05).

**Fig 2:**
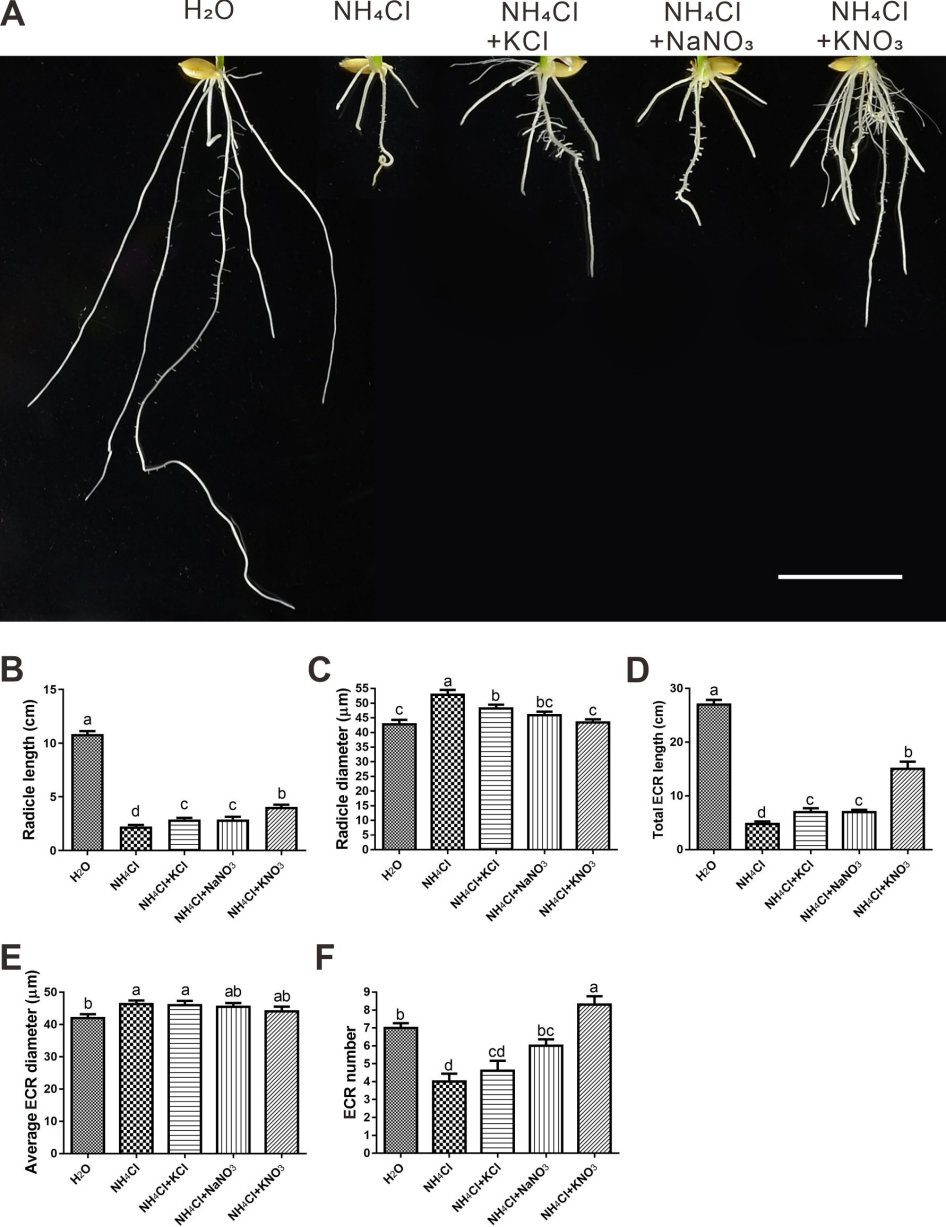
Root architecture. (A) Photograph of roots grown for 7 days. Scale bar = 2 cm. (B-F) Measured parameters. ECR = embryonic crown root. Data are mean ± SD, n = 10; t-tests were used to identify significant differences, and different letters represent significant differences among different treatments (p < 0.05).

**Fig 3:**
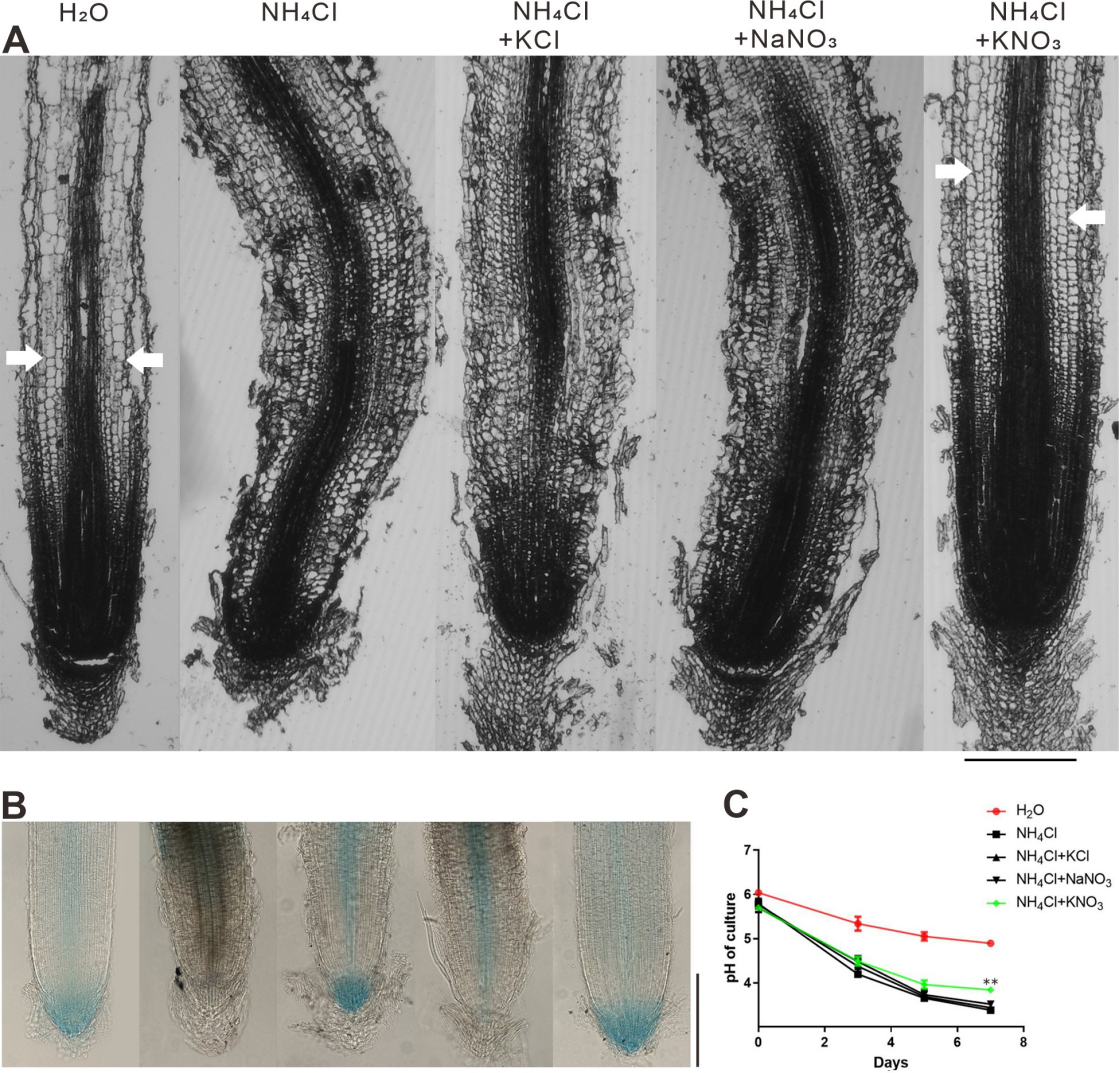
Morphology, auxin responsiveness, and rhizosphere acidification. (A) Micrographs of paraffin sections of radicles from 7-day-old seedlings. The approximate boundary of the elongation zone is marked by white arrowheads. (B) Micrographs of 7-day-old roots expressing DR-5::GUS, stained for GUS activity. In A and B, roots shown are the roots representative, selected from 10 seedlings in each treatment. Scale bars = 50 μm. (C) The pH of the culture solutions was measured every 2 days from the 3^rd^ day after seed germination. Data are the mean ± SD; t-tests were used to identify significant differences; ** (p < 0.01) represent significant differences between each alleviation treatment and ammonium toxicity treatment.

We found that ammonium toxicity in the roots of *Oryza sativa japonica ZH11* seedlings was alleviated to a much greater extent when potassium and nitrate were supplied together than when they were supplied separately (Fig 1A and 1B). We tried various concentrations (from 0.5 to 12 mM) of NH_4_Cl, and found that the 4 mM NH_4_Cl is a propriate concentration to generate enough ammonium toxic effect on rice seedling roots while facilitate sampling (S1A Fig). We also tried different concentration ratios of alleviative ions versus toxic ion in the 12 mM NH_4_Cl (S1B Fig), and found that the differences among 5 treatments are consistent with that in Fig 1C. Therefore, we speculated that there might be a synergistic mechanism by which potassium and nitrate participate in ammonium toxicity alleviation. To test our hypothesis, we first determined that neither sodium nor chlorine had any effect on ammonium toxicity and its alleviation (Fig 1B–1D). The combined application of potassium and nitrate had significantly stronger alleviation effects on embryonic crown root (ECR) length than individual applications, while the effects on radical length, crown root number and root diameter seemed additive (Fig 2). The morphologies and auxin distributions of the radicles and the embryonic crown roots in each treatment were consistent. Under ammonium toxicity treatment, the root cap was enlarged and grew abnormally, which was ameliorated only by KNO_3_ (Fig 3B). We can see cells which are beginning to elongate (a sign indicating the beginning of the elongation zone) in the H_2_O and KNO_3_ alleviation treatments, but we cannot see this kind of cell in the other 3 treatments (Fig 3A). The cells in the cortex of the control and KNO_3_ alleviation treatment were similar and rectangular, while cells in the cortex of the ammonium toxicity and KCl or NaNO_3_ alleviation treatments were elliptical and irregular (Fig 3A).

DR5 is a synthetic auxin response element which reports on auxin responsiveness in a DR5:GUS transgenic plant. Under ammonium toxicity, the auxin signal in the rice seedling root tips was markedly weakened compared with that in the control (Fig 3B). Comparing to the control, KCl treatment partly recovered the distribution of the GUS signal in the root cap, and slightly reduced the GUS signal in the meristem zone. NaNO_3_ treatment could not restore the GUS signal in the root cap and the meristem zone (Fig 3B). In the case of KNO_3_, the distribution of the GUS signal was essentially the same as that in the control (Fig 3B), indicating that KNO_3_ had a better alleviating effect on auxin responsiveness. Rhizosphere acidification caused by ammonium toxicity was lessened by only KNO_3_ and not by KCl or NaNO_3_ (Fig 3E).

Therefore, in terms of the biomass, root tip cell morphology, and rhizosphere acidification, it appears that potassium and nitrate act synergistically to alleviate ammonium toxicity in rice roots.

### Ammonium uptake and assimilation

**Fig 4:**
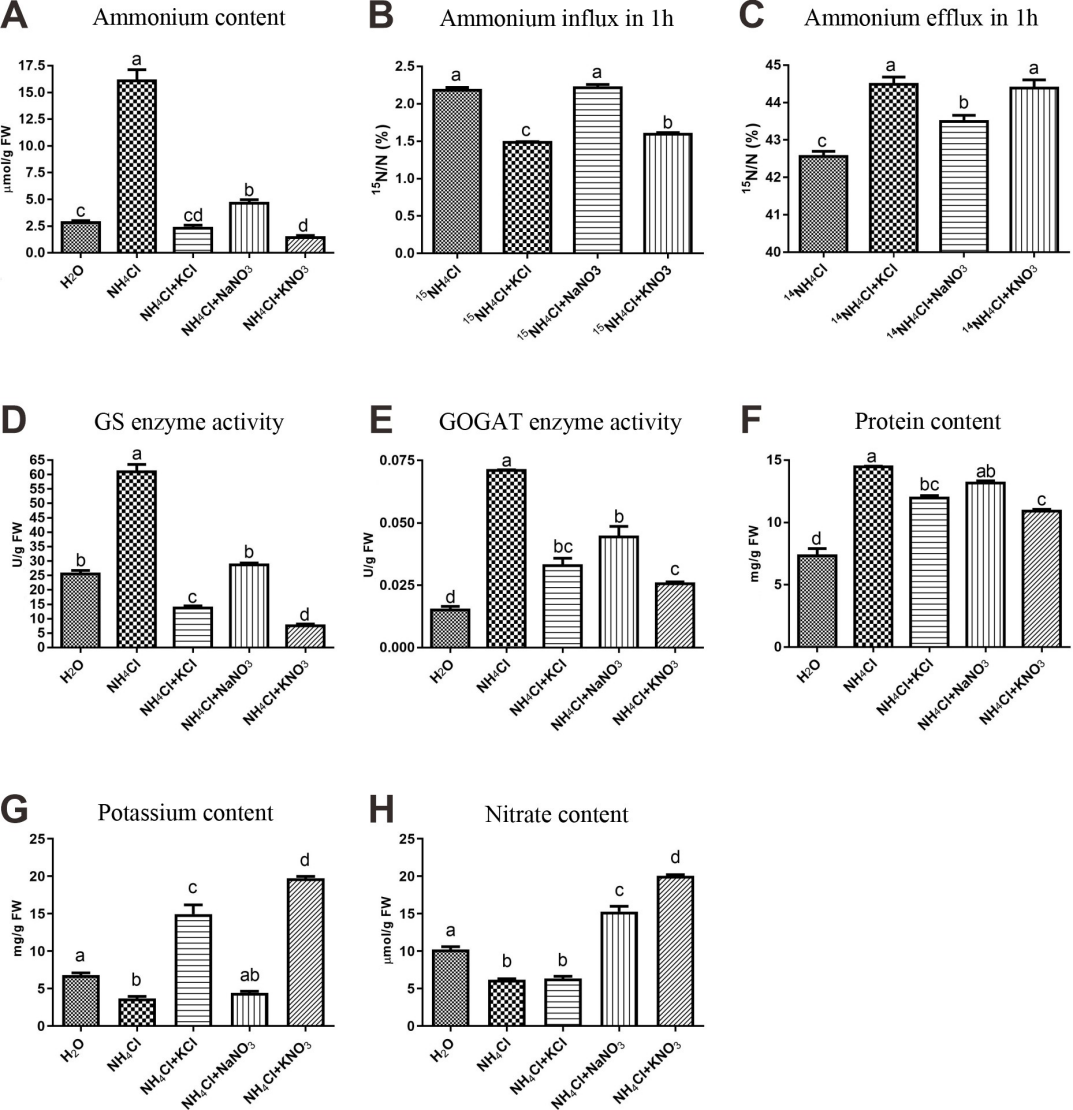
Composition, transport, and enzyme activity related to nitrogen status. (A) The ammonium concentrations. (B and C) For the ^15^NH_4_^+^ transport assay, rice seedlings were grown for 7 days in ^14^NH_4_Cl (for B) or ^15^NH_4_Cl (for C) solution containing no other substance and then transferred to solutions containing ^15^NH_4_Cl (for B) or ^14^NH_4_Cl (for C) with the 3 alleviation treatments for 1 hour (see Materials and methods) before the root ^15^N content was measured. (D-H) Data are mean ± SD; t-tests were used to identify significant differences, and different letters represent significant differences among different treatments (p < 0.05).

To analyze how potassium and nitrate alleviated rice ammonium toxicity, we measured the ammonium content in roots. The ammonium content under the ammonium toxicity treatment was the highest among all treatments (Fig 4A). Both KCl and KNO_3_ reduced the ammonium content to a level comparable to that of the control, while the alleviation effect of NaNO_3_ was somewhat weaker (Fig 4A). These results suggested that both potassium and nitrate alleviated ammonium toxicity by reducing the net influx of ammonium. Furthermore, we compared the effects of potassium and nitrate on root ammonium influx and efflux using ^15^N-labeled NH_4_Cl. Potassium reduced ammonium influx and efflux, while nitrate reduced only ammonium efflux (Fig 4B and 4C). However, consistent with the ammonium content, the impacts of KCl and KNO_3_ on root ammonium influx and efflux were largely the same, suggesting that the differences in ammonium toxicity alleviation between KCl and KNO_3_ could not be attributed only to the regulation of ammonium content. In addition, the GS and GOGAT enzyme activities were similar to ammonium content (Fig 4D). In contrast, protein levels were elevated in all of the treatments although somewhat less so by KNO_3_ (Fig 4F).

We also measured the potassium and nitrate levels in roots (Fig 4G and 4H). As expected, ammonium treatment reduced concentrations of both potassium and nitrate (by about 50%); also as expected, provision of either ion raised the level in the water control. However, the level of each ion in the root was increased even more in the combined treatment than in the single treatment, suggesting metabolism of the ions is linked.

### Analysis of differentially expressed genes

**Fig 5:**
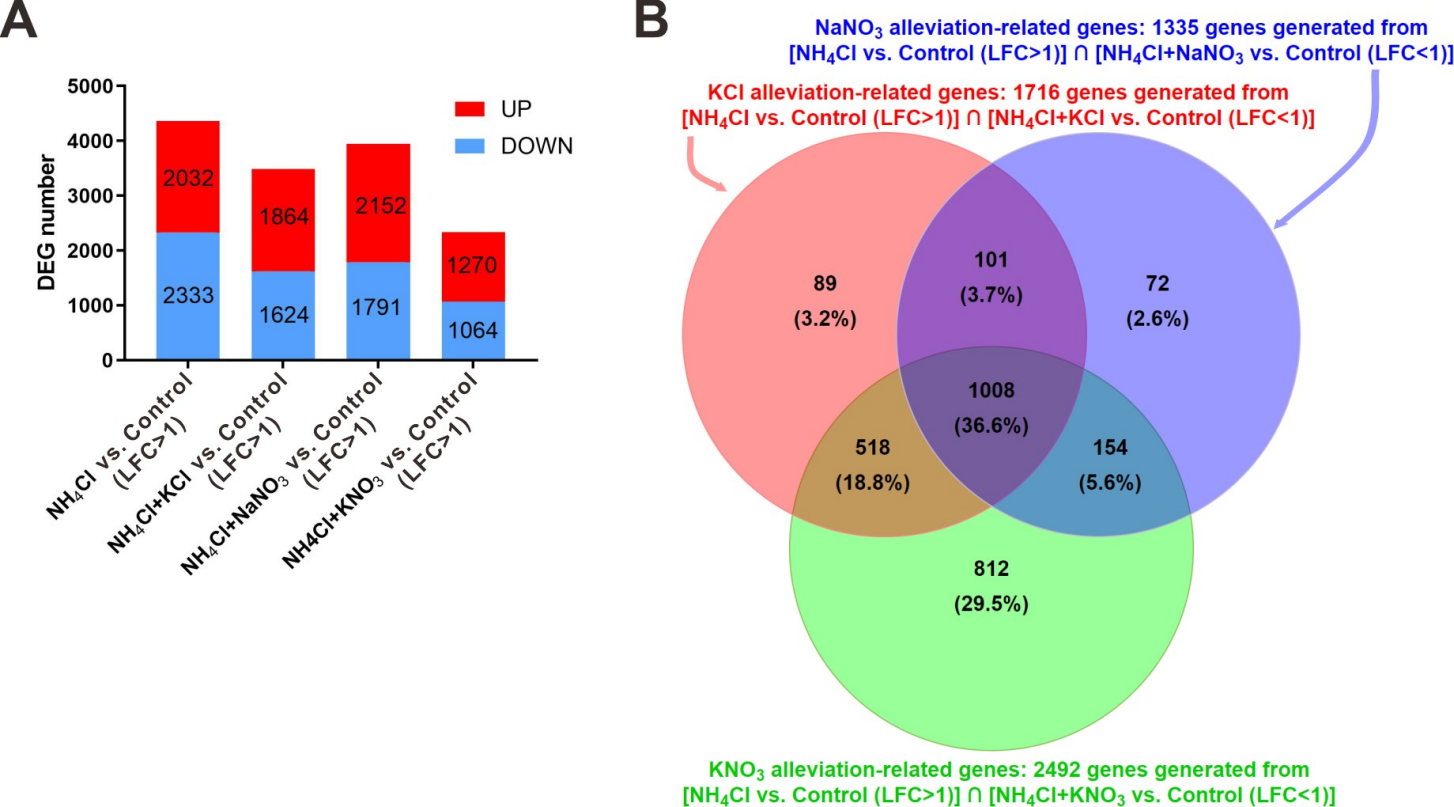
Differentially expressed genes (DEGs). (A) Numbers of differentially expressed genes between given treatments and the control (H_2_O). (B) Venn diagram showing alleviation-related gene numbers in each alleviation treatment. The expression of these genes was differentially regulated by ammonium toxicity and restored to the control level by the 3 alleviation treatments.

Whole-transcriptome analysis revealed that the number of differentially expressed genes (|log2FC| > 1 and adjusted p value < 0.05) in each treatment compared with the control was related inversely to the same comparison for root biomass (Figs 1C, 1D and 5A). From these differentially expressed genes, we arbitrarily selected 24 genes that appeared in all 4 groups to detect their expression level by quantitative real-time polymerase chain reaction (qRT-PCR), and all 24 gene expression differences among the 5 treatments were consistent with the corresponding fragments per kilobase per million (FPKM) values generated from the transcriptome analysis, verifying the validity of the RNA-Seq analysis results (S2 Fig).

We defined 4365 differentially expressed genes between the NH_4_Cl treatment and the control as toxicity-related genes (Fig 5A). To further investigate the unique role of KNO_3_ in the alleviation of ammonium toxicity, we compared the number of genes among the toxicity-related genes with expression that returned to levels comparable to that in the control (|log2FC| < 1 and adjusted p value < 0.05) under different alleviation treatments. There were 1716, 1335, and 2492 genes that met these requirements in the differentially expressed gene groups of the KCl, NaNO_3_, and KNO_3_ treatments, respectively (Fig 5B). These genes were treated as alleviation-related genes. There were 812 genes with expression that could only be recovered under KNO_3_ alleviation (Fig 5B, S1 Table, S3 Fig).

We found 3 proton exchanger/antiporter genes (OS05G0113300, OS03G0828600, OS07G0666900) among the KNO_3_-specific alleviation-related genes (812 genes), and these genes might play a role in the specific mechanisms that alleviate rhizosphere acidification. To gain further insight into the possible processes involved in ammonium toxicity and alleviation, we examined the distributions of toxicity-related genes and alleviation-related genes based on pathway enrichment (KEGG) analysis (S4A Fig) and gene ontology (GO) classification (S4B Fig). The KEGG and GO results suggest that the combined application of potassium and nitrate generated alleviation via changes in gene expression related to stress responses, redox states, and metabolism.

### Weighted gene correlation network analysis

**Fig 6:**
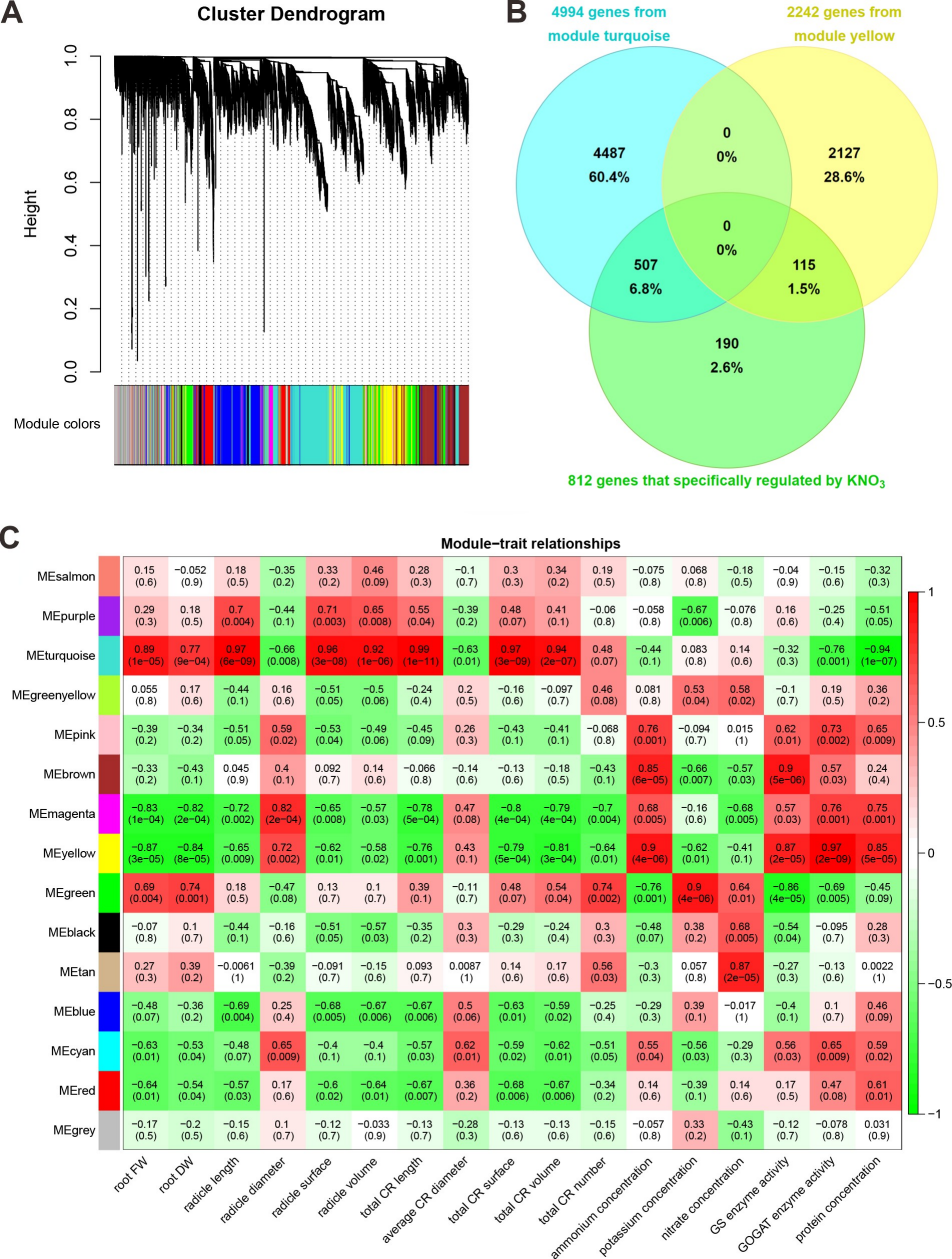
The weighted gene correlation network analysis. (A) Hierarchical cluster tree showing coexpression modules identified by WGCNA. Each leaf in the tree represents 1 gene. The major tree branch comprises 15 modules labeled by different colors. (B) Venn diagram showing the interactions of genes in root-trait coupled modules and differentially expressed genes with expression that was recovered only by KNO_3_ under ammonium toxicity. (C) Module-trait association. Each row corresponds to a module. Each column corresponds to a trait. The color of each cell at the row-column intersection indicates the correlation coefficient from -1 (green) to 1 (red).

To characterize the gene expression changes that occurred under different treatments, we clustered the expression patterns by the WGCNA method, leading to the identification of 15 distinct WGCNA modules (Fig 6A and 6C). Analysis of the module-trait relationships revealed that the ‘turquoise’ module was highly positively correlated with root biomass and all root traits except for root diameter (Fig 6C). The ‘turquoise’ module was also highly negatively correlated with GOGAT enzyme activity and protein concentration (Fig 6C). Therefore, the genes of the ‘turquoise’ module were considered to play an important role in root growth. The ‘yellow’ module was highly positively correlated with the GOGAT enzyme activity, protein concentration, and ammonium concentration (Fig 6C). The genes of the ‘yellow’ module might be negatively associated with root growth.

There were 507 genes in the ‘turquoise’ module and 115 genes in the ‘yellow’ module with differentially regulated expression in response to ammonium toxicity that was specifically recovered by KNO_3_ (Fig 6B). These genes with expression that was highly correlated with traits might be responsible for the KNO_3_-specific alleviation effect on root growth. We used a module membership (MM) > 0.9 and a gene significance (GS) > 0.8 as parameters to identify hub genes among the 507 genes, and we found 62 hub genes (S2 Table). The coexpression network was visualized by Cytoscape with a WGCNA edge weight > 0.35 (S4C Fig). Several genes involved in cell wall formation were identified in the network, such as genes associated with cellulose synthesis (OsCesA4, OsCesA7, OsCesA9), lignin catabolic process (Os01g0850700), cell wall modification (OsEXPB5), and cell wall proteins (OsFLA11, OsFLA27) (S2 Table). These results suggested that under ammonium toxicity, KNO_3_ specifically restores the expression of genes related to cell wall formation and alleviates the effects of ammonium toxicity on cell morphology and root growth by restoring cell wall formation.

### A cell wall regulation network related to this synergism

**Fig 7:**
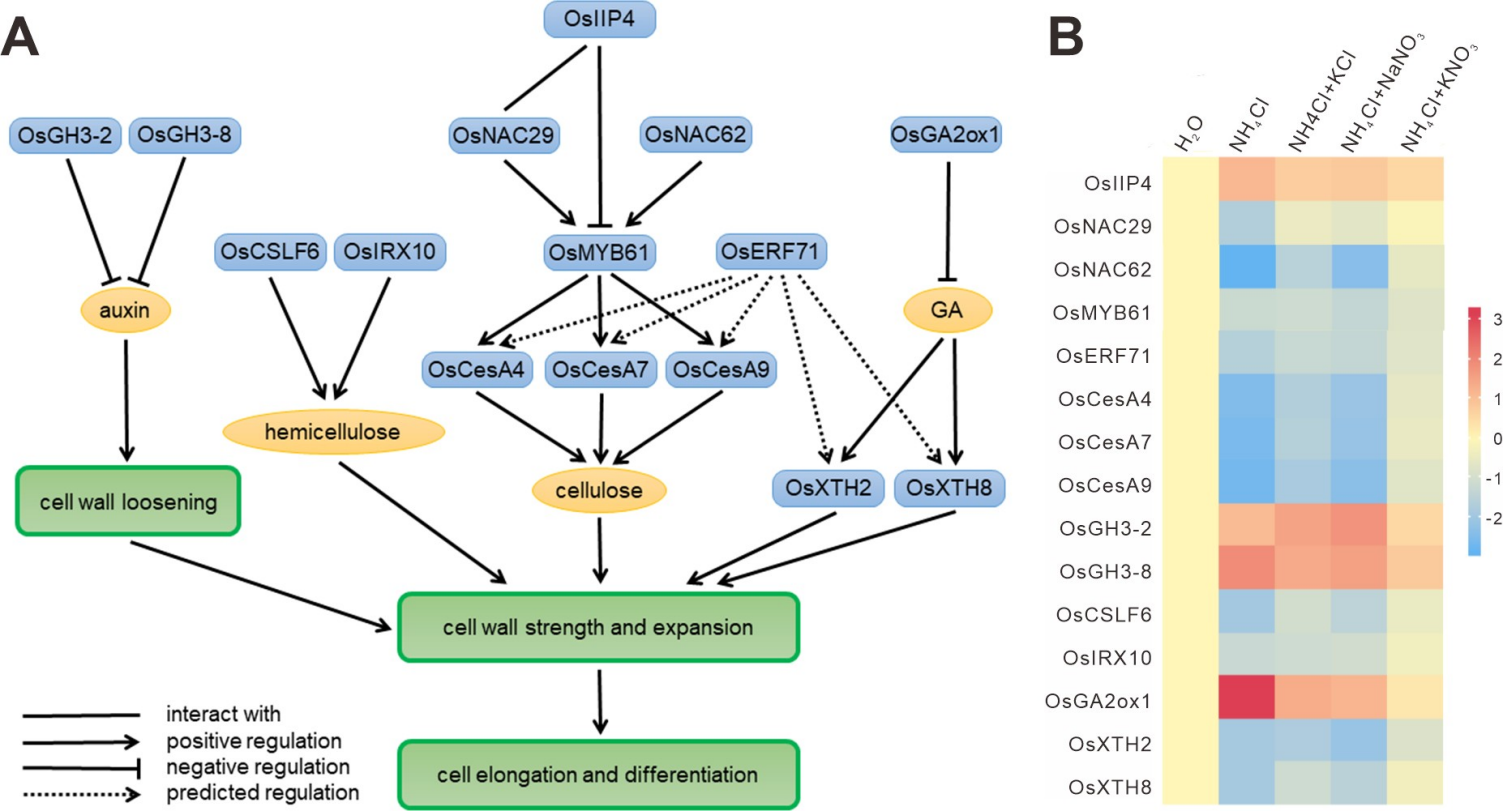
The cell wall regulation network related to this synergism. (A) A network involved in cell wall formation was constructed according to the literature (line) and predicted transcriptional regulation (dashed line). Blue boxes indicate genes or proteins; yellow ovals indicate chemicals; green boxes indicate biological processes. (B) Expression patterns of genes in this network under the treatments.

Based on these findings, we re-examined the expression of genes associated with root cell formation. Cellulose is an important component of the cell wall. Analysis of the transcription regulation network for cellulose synthesis suggests that OsNAC29 and OsNAC62 directly regulate OsMYB61, which in turn activates the expression of cellulose synthase genes (CESAs), such as OsCESA4, OsCESA7, and OsCESA9 [29]. The expression of OsMYB61 and OsCESAs is negatively regulated by OsIIP4, which does not bind their promoters and interacts with OsNAC29 [30]. Our results showed that the expression of these genes was repressed under NH_4_Cl treatment, and KNO_3_ resulted in better recovery of the expression of these genes (Fig 7). Furthermore, similar results were observed with OsCSLF6 and OsIRX10 (Fig 7), which are involved in the hemicellulose synthesis pathway. Thus, the cellulose and hemicellulose synthesis pathways mentioned herein might be a regulatory mechanism induced by KNO_3_.

Xyloglucan endotransglucosylases/hydrolases (XTHs) play an important role in the construction of the primary cell wall by mediating the cleavage and rejoining of β-(1–4)-xyloglucans. There were 2 XTH genes (OsXTH8 and OsXTH2) among the 507 genes. OsXTH8 is upregulated by gibberellin and is highly expressed in vascular bundles of the leaf sheath and young roots, where cells are actively undergoing elongation and differentiation [31]. OsXTH2 is also upregulated by gibberellin [32]. Gibberellin can be converted to nonbiologically active gibberellin in plants by 2β-hydroxylation, a reaction catalyzed by gibberellin 2-oxidase. OsGA2ox1 encodes a gibberellin 2-osidase, which is functional in vitro and in vivo [33]. Our results showed that KNO_3_ restored or nearly so the expression levels of OsXTH8, OsXTH2, and OsGA2ox1 under NH_4_Cl toxicity (Fig 7). This result suggests that the gibberellin metabolic pathway mentioned herein was disrupted by ammonium toxicity, leading to abnormal root cell elongation, which could be effectively recovered by the synergistic effects of KNO_3_.

Auxin is known to stimulate cell elongation by increasing cell wall extensibility. The OsGH3 protein controls auxin homeostasis by catalyzing the conjugation of IAA to amino acids. Overexpression of OsGH3-2 reduces the free IAA level [34], and downregulation of OsGH3-8 leads to an auxin overproduction phenotype [35]. Our results again showed that the expression levels of OsGH3-2 and OsGH3-8 were upregulated by NH_4_Cl treatment, and high expression was maintained under the KCl and NaNO_3_ treatments (Fig 7). Only the KNO_3_ treatment restored the expression of these 2 genes to the control level, which was consistent with the auxin distribution result indicated by DR5:GUS (Fig 3B). Thus, the synergistic effects might also regulate the auxin pathways to result in enhanced beneficial effects on rice roots under ammonium treatment.

## Discussion

### Potassium and nitrate might have a synergistic effect on ammonium toxicity alleviation in rice roots

In 4 mM NH_4_Cl solution, the growth of rice seedling roots was severely hampered, which is a typical ammonium toxicity. It is widely known that supplementation with nitrate or potassium alleviates ammonium toxicity in plants [1, 5, 17]. To exclude the interference of other substances on the alleviation effect of potassium and nitrate on rice ammonium toxicity, we did not use any culture media in this research. In rice, the endosperm can provide sufficient nutrition for seedling growth over 1 week after germination. We found that the addition of KNO_3_ resulted in a significantly enhanced promotion of root growth compared with that of KCl or NaNO_3_.

The rhizosphere acidification is the most typical characteristics of plant ammonium toxicity [36]. We found that only the combined treatment of potassium and nitrate reduced the level of rice rhizosphere acidification caused by ammonium toxicity. In the roots of KNO_3_ treatment, there were more nitrate uptake and less ammonium uptake compared to that of the NaNO_3_ treatment under ammonium toxicity. This might help to alleviate the root rhizosphere acidification level in the KNO_3_ treatment. Our study showed that the ammonium toxicity notably decreased the number of embryonic crown roots, while the KNO_3_ treatment increased their number to even more than that of the control (Fig 2F). In rice in general, the number of embryonic crown roots is 5. Considering that the number of embryonic seminal roots in maize is variable [37], we speculate that the number in rice might be altered by certain conditions. Evidently, ammonium toxicity and the combined treatment of potassium and nitrate alter embryonic crown root number in rice. Additionally, roots in the KNO_3_ treatment are shorter and thicker than those of the control, though there is no biomass difference between them. We suggest that the cause of this phenomenon is related to the nutrient status in these two treatments.

The nitrogen status regulates cell division and expansion and thus shapes architecture [38]. Ammonium inhibits cell elongation rather than proliferation in *A*. *thaliana* roots [4]. The inhibitory effect of ammonium on *A*. *thaliana* primary root elongation was mainly achieved by reducing the length of the meristem and elongation zones, although elemental elongation rate was also inhibited [6]. Our results show that ammonium toxicity causes an irregular cell morphology, seemingly impaired cell elongation, and loss of auxin signaling in rice seedling roots. KNO_3_ treatment effectively recovered the root cell morphology, resulting in a regular cell arrangement and a recovered auxin distribution that was almost the same as that of the control.

### Potassium-nitrate synergism might regulate gene expression associated with root growth and ammonium assimilation

Ammonium causes changes in gene expression in roots, which in turn leads to a series of changes in growth [36]. Our results show that potassium and nitrate restore the expression of some genes to levels comparable to those in the control. Therefore, the restoration of the expression of these genes represents an intrinsic mechanism of ammonium toxicity alleviation by these 2 ions. We identified 812 genes with expression that was specifically restored by KNO_3_ but not by KCl or NaNO_3_, suggesting that the synergism between potassium and nitrate might activates specific transcriptional regulation to alleviate ammonium toxicity in rice roots.

Coexpression network analysis helps investigate thousands of genes with identical expression patterns. It is an important method for gene interaction analysis and the interpretation of molecular mechanisms [39]. In this study, we identified a “turquoise” module that was highly positively correlated with almost all root traits (excluding root diameter), and a “yellow” module that was highly positively correlated with tissue ammonium content and ammonium assimilation. The correlation analysis of the traits showed a negative correlation between most root traits and ammonium assimilation. Therefore, we hypothesized that gene expression in these 2 modules was positively and negatively correlated with root growth. Among the 812 alleviation-related genes with expression that was specifically regulated by KNO_3_, 507 genes belonged to the ‘turquoise’ module, and 115 genes belonged to the ‘yellow’ module. Therefore, the specific synergistic alleviation effect of KNO_3_ on root ammonium toxicity might be mainly achieved by regulation of the expression of these genes. No significant enrichment of GO terms was found in the 115 genes. The 507 genes were predominantly enriched in GO terms such as oxidation reduction, carbohydrate metabolic process, lignin catabolic process, and response to oxidation stress. Furthermore, we identified 62 hub genes from the 507 genes. MapMan analysis [40] of these hub genes showed that they were enriched in 3 categories: genes associated with cell wall formation, various enzymes, and transporters. Some genes were significantly associated with root growth traits and cell wall formation, so changes in their expression could result in different morphologies of rice root tip cells under ammonium toxicity or different alleviation conditions.

### This synergism can alleviate rice root ammonium toxicity by regulating root cell wall formation

With changes in external nutrients, the composition of the cell wall determines the morphology of root cells and tissues, further influencing plant growth [41]. Although there is growing evidence that the reprogramming of cell wall genes is critical for plant adaptation to nutritional status, an understanding of the molecular mechanisms that control these changes is now emerging. In crops, nitrogen status affects the mechanical strength and disease resistance of stems, which are regulated by the cell wall organization and strength, suggesting that the cell wall structure is regulated by nitrogen status [41, 42]. The expression of genes associated with lignin and cellulose synthesis is significantly downregulated when rice is grown in a high-nitrogen environment [42, 43]. Consistent with this phenomenon, a high-nitrogen growth environment causes a significant decrease in cellulose and lignin in the rice root cell wall, while nitrogen deficiency causes an increase in cellulose in rice roots [44].

Based on differentially expressed gene analysis and WGCNA, we revealed a transcriptional regulatory network of genes associated with rice root cell wall formation that are affected by nutritional status (Fig 4, S3 Table). Previously, the importance of the changes in the *A*. *thaliana* cell wall in response to ammonium stress has been reported [45]. These authors found that the ammonium-mediated inhibition of growth is related to a more rigid cell wall structure which limits the expansion of cells, and identified some genes as important contributors to changes in the cell wall of leaves [45]. Additionally, in the roots of the legume *Medicago truncatula*, Royo et al. (2019) showed that the higher expression of the enzymes of the phenylpropanoid metabolism during ammonium nutrition is accompanied with a higher content of lignin, confirming reinforcement of the cell wall as a tolerance mechanism of [46]. Among our toxicity related genes, we found putative orthologues for all of the cell wall modifying proteins mentioned by Podgórska et al. (2017) [45]. And most of these orthologous genes, specifically, pectin lyase, polygalacturonase, peroxidase, xyloglucan endotransglycosylase genes also appeared in our KNO_3_-specific alleviation-related genes (S4 Fig). We even found 2 XTH genes (OsXTH2 and OsXTH8) in our regulation network of the root cell wall formation (Fig 7). Likewise, similar to the findings of Royo et al. (2019) [46], we identify as related to ammonium toxicity many of the same genes, including genes involved in metabolism of phenylpropanoids, amino acids, carbohydrates, flavonoids, diterpenoids, antioxidants, and numerous membrane protein genes such as integral and intrinsic protein genes, various ion binding protein and transporter genes, and many peroxidase genes. The genes involved in metabolisms of glyoxylate, dicarboxylate, glycine, serine, threonine, glutathione, flavonoid, and the genes of integral and intrinsic to membrane were also appeared in our KNO_3_-specific alleviation-related genes. Additionally, many cellulose metabolism genes only differently expressed in the KNO_3_ alleviation treatment (they are not a DEG in the other 4 treatments) (S4 Fig). These findings suggest that cell wall formation in rice roots is related not only to nitrogen status but also to interactions between different nutrients. The separate addition of nitrate and potassium into the ammonium growth environment hardly alleviated the damage to cell wall formation, while the mixture of nitrate and potassium restored the expression of genes related to cell wall synthesis, and evidently restored cell wall formation. Moreover, these processes also involve hormonal pathways such as the auxin and gibberellin pathways. These results suggest that the effects of nitrogen on cell wall formation involve not only the morphological impact of nitrogen itself (ammonium or nitrate) but also the synergism between nitrogen and other nutrients (potassium). This phenomenon is also consistent with the knowledge that, in agricultural practices, mixed fertilizer application results in better crop growth performance.

We found 2 transcription factors in the KNO_3_-specific alleviation-related genes (Os03g0820300 and Os12g0582600). These genes might play a role in regulating nitrogen use-associated genes, thereby contributing to nitrogen use efficiency [47]. Rice is a member of the *Gramineae*, a plant that is a model organism for most crops for human consumption. An in-depth study of these mechanisms will reveal the link between environmental nutrition and crop growth and provide a theoretical basis for resolving the conflict between high yield and poor disease resistance in crops by improving the fertilizer utilization efficiency [38, 47].

## Supporting information

S1 Fig Supplementary data of the root growth. (A) 5-day-old roots of rice seedlings under 0/0.5/1/2/4/8/12 mM (from left to right) NH_4_Cl. (B) The fresh weight of roots under different treatments. The concentration ratio of “alleviative ions”: “toxic ion” = 1: 2, 1: 1, 2: 1 in 12 mM concentration.
(TIF)

S2 Fig The validity of the RNA-Seq analysis results. The relative expression patterns among 5 different treatments of 24 DEGs (randomly selected from all DEGs that appeared in all 4 DEG groups) were consistent with the corresponding relative FPKMs generated from transcriptome analysis.
(TIF)

S3 Fig Expression heatmap of the clustered genes. The gene expression heatmap of 10 groups of clustered KNO_3_-specific alleviation-related genes. The color of each cell indicates the Log_2_FC of genes comparing to control (H_2_O) from -6 (blue) to 6 (red).
(TIF)

S4 Fig KEGG, GO and coexpressed hub genes. (A) KEGG pathway enrichment analysis of DEGs. (B) GO enrichment analysis of DEGs. (C) Cytoscape presentation of coexpressed hub genes among 507 genes, with the criterion of a module membership > 0.9 and a gene significance > 0.8.
(TIF)

S1 Table The Log_2_FC of the KNO_3_-specific alleviation-related genes. The order of the genes in this table is consistent with that in the heatmap (S3 Fig).
(CSV)

S2 Table Sixty-two hub genes with expression that could be recovered by specific synergism. (XLSX)

S3 Table Hub genes in the cell wall construction network that were regulated by specific synergism. (XLSX)

S1 File Supporting methods. (PDF)
