# Supplementary figures and images for: Evidence that synergism between potassium and nitrate enhances the alleviation of ammonium toxicity in rice seedling roots
Source: PLoS One. 2021 Sep 9;16(9):e0248796. doi: 10.1371/journal.pone.0248796 (PMC8428561; doi:10.1371/journal.pone.0248796)

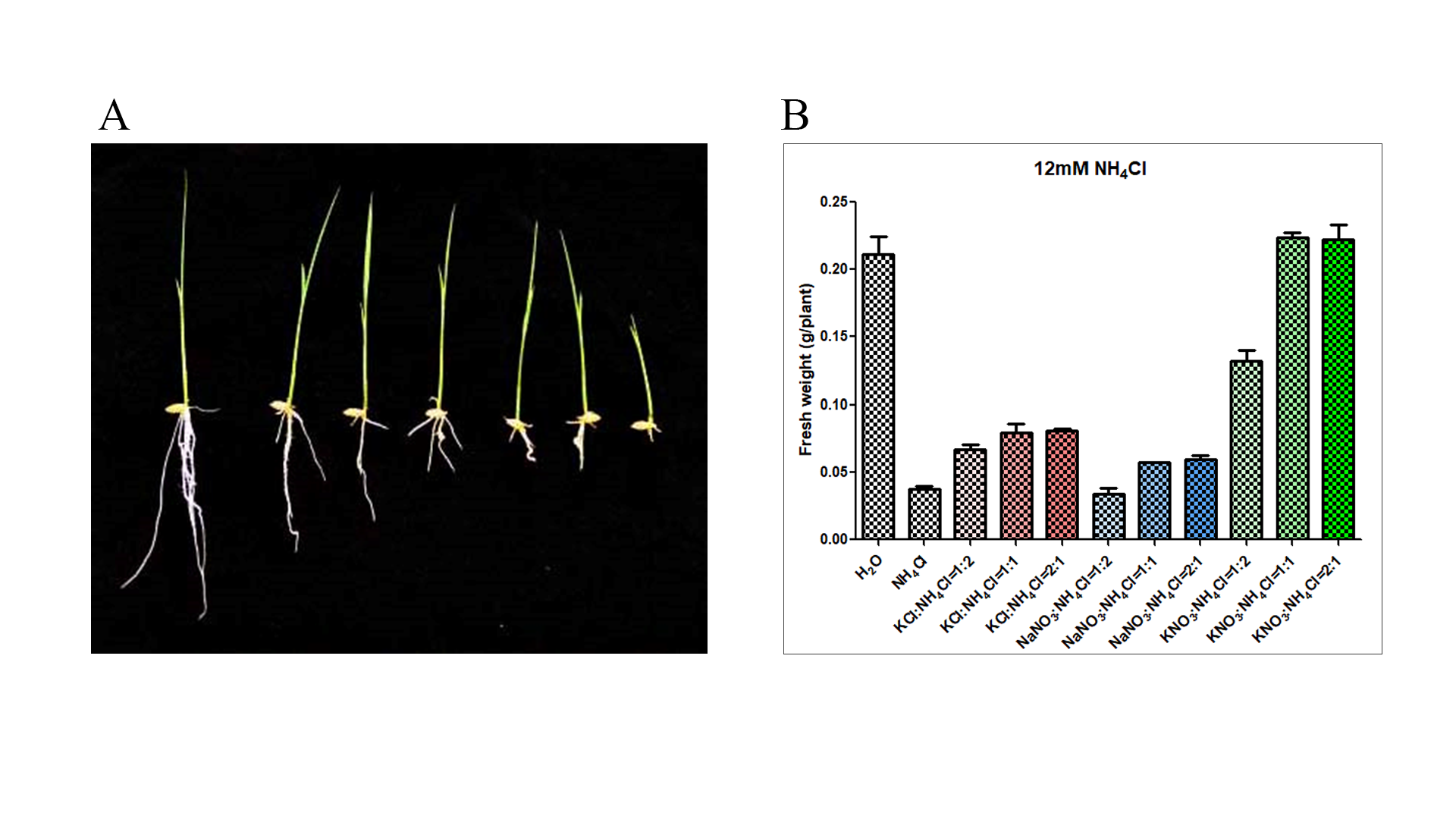

Supplement: S1 Fig — (A) 5-day-old roots of rice seedlings under 0/0.5/1/2/4/8/12 mM (from left to right) NH4Cl. (B) The fresh weight of roots under different treatments. The concentration ratio of “alleviative ions”: “toxic ion” = 1: 2, 1: 1, 2: 1 in 12 mM concentration. (TIF) [file pone.0248796.s001.tif]

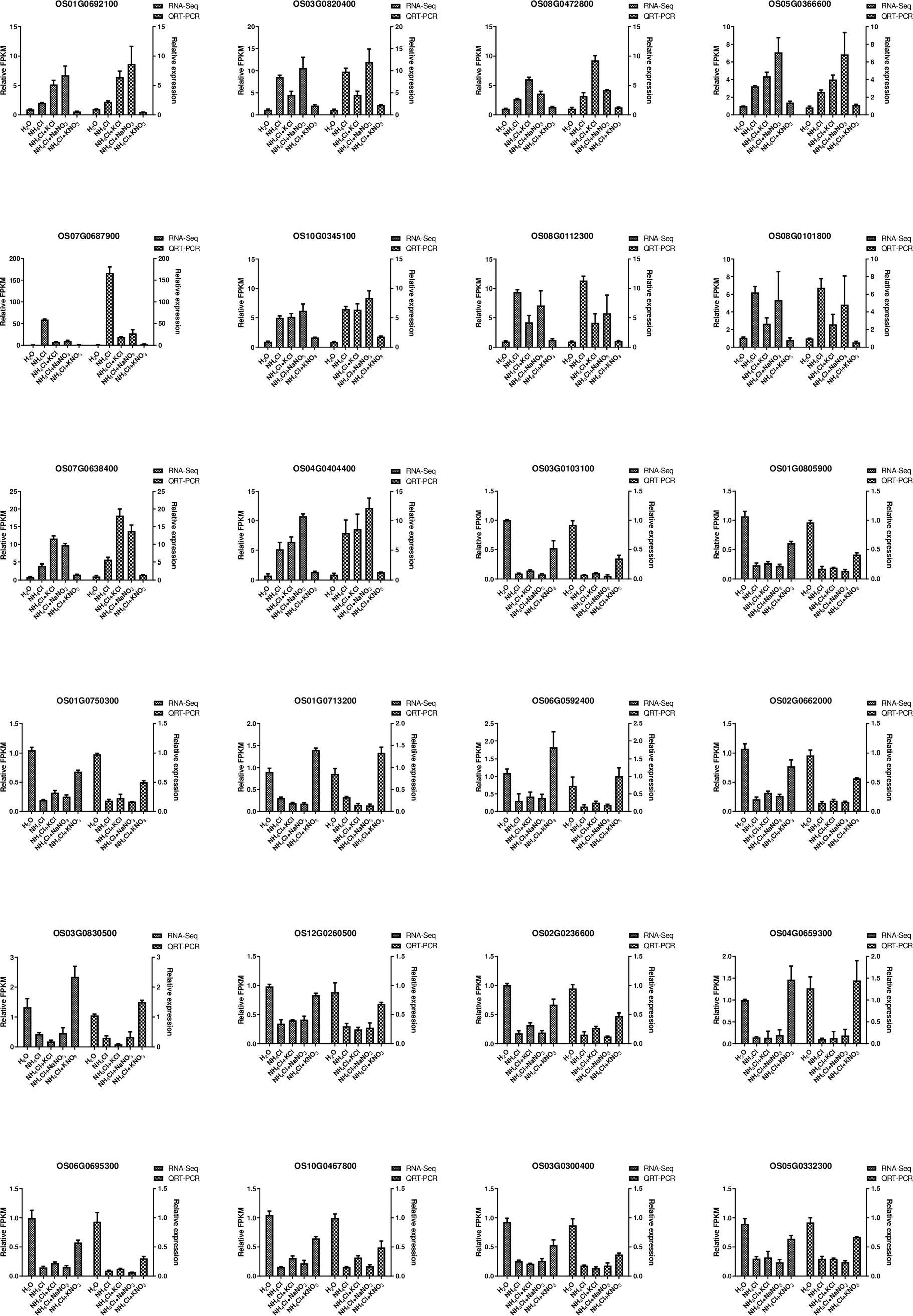

Supplement: S2 Fig — The relative expression patterns among 5 different treatments of 24 DEGs (randomly selected from all DEGs that appeared in all 4 DEG groups) were consistent with the corresponding relative FPKMs generated from transcriptome analysis. (TIF) [file pone.0248796.s002.tif]

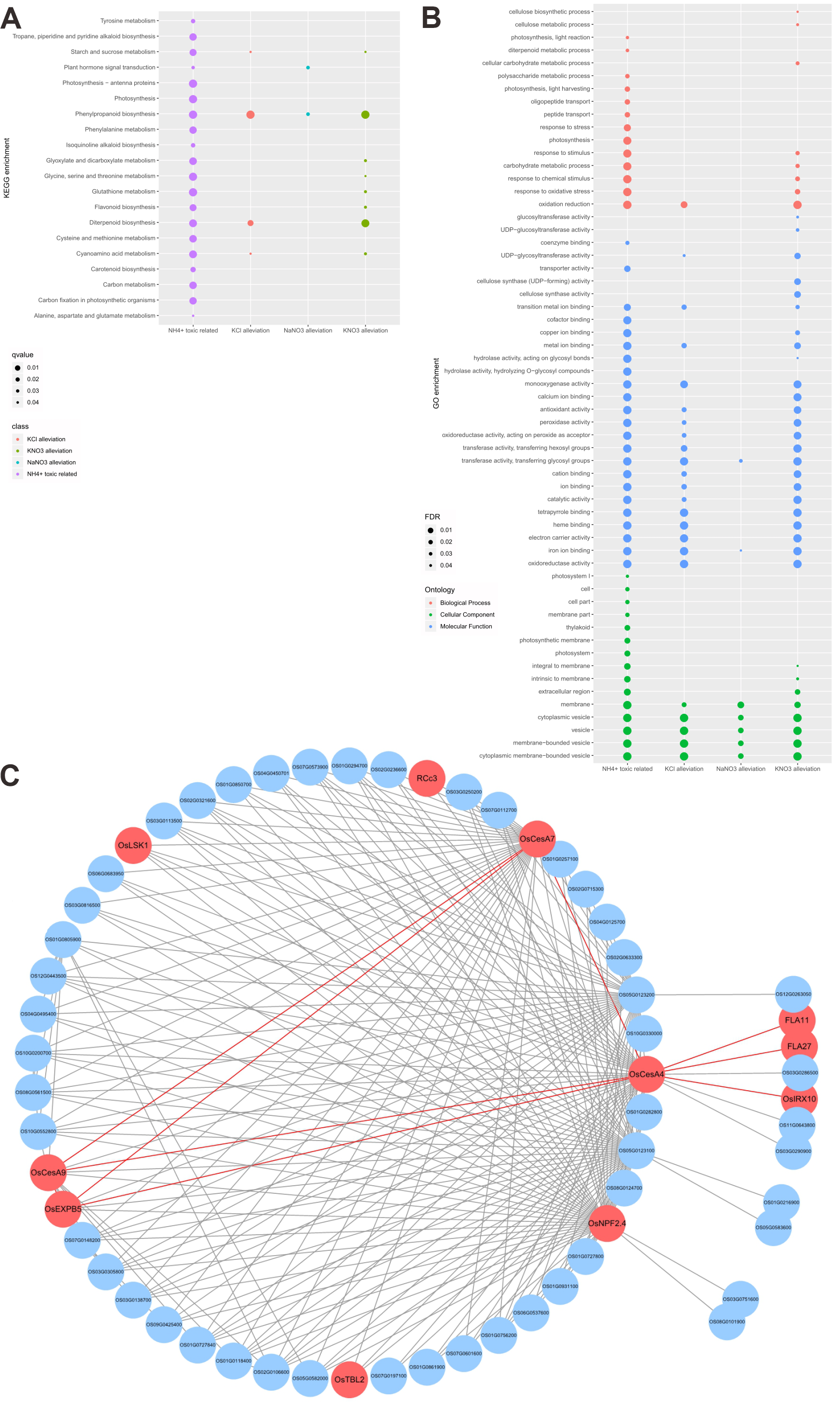

Supplement: S4 Fig — (A) KEGG pathway enrichment analysis of DEGs. (B) GO enrichment analysis of DEGs. (C) Cytoscape presentation of coexpressed hub genes among 507 genes, with the criterion of a module membership > 0.9 and a gene significance > 0.8. (TIF) [file pone.0248796.s004.tif]
