# Supplementary material for: Evidence that synergism between potassium and nitrate enhances the alleviation of ammonium toxicity in rice seedling roots
Source: PLoS One. 2021 Sep 9;16(9):e0248796. doi: 10.1371/journal.pone.0248796 (PMC8428561; doi:10.1371/journal.pone.0248796)
Supplement: S1 File — (PDF) [file pone.0248796.s008.pdf]

Supplementary methods

qRT-PCR:

Roots were homogenized in liquid N_2. Total RNA was extracted using TRIzol Reagent (Invitrogen, Thermo Fisher Scientific, USA); then, the residual DNA was digested with DNase I (Promega, USA). cDNA was synthesized using M-MLV reverse transcriptase (Promega, USA). qRT-PCR was performed using a CFX96 Real-Time PCR Detection System (Bio-Rad, USA) with iQ SYBR Green Supermix (Bio-Rad, USA) for 40 cycles (95°C for 10 s, 60°C for 10 s, 72°C for 15 s). EF-1α, UBQ5, and ARF [1] were used as internal references. The data were derived from 3 technical replications.

GO and KEGG analysis:

GO enrichment analysis was completed using AgriGO V2.0 [2]. KEGG analysis was performed using the R package clusterProfiler [3]. The GO and KEGG graphs were developed using ggplot2 [4].

References:

1. Wang Z, Wang Y, Yang J, Hu K, An B, Deng X, et al. Reliable Selection and Holistic Stability Evaluation of Reference Genes for Rice Under 22 Different Experimental Conditions. Applied Biochemistry and Biotechnology. 2016;179(5):753-

75. doi: 10.1007/s12010-016-2029-4.

2. Tian T, Liu Y, Yan H, You Q, Yi X, Du Z, et al. agriGO v2.0: a GO analysis toolkit for the agricultural community, 2017 update. Nucleic Acids Res. 2017;45(W1):W122-W9. Epub 2017/05/05. doi: 10.1093/nar/gkx382. PubMed PMID: 28472432; PubMed Central PMCID: PMCPMC5793732.

3. Yu G, Wang LG, Han Y, He QY. clusterProfiler: an R package for comparing biological themes among gene clusters. OMICS. 2012;16(5):284-7. Epub 2012/03/30. doi: 10.1089/omi.2011.0118. PubMed PMID: 22455463; PubMed Central PMCID: PMCPMC3339379.

4. Ginestet C. ggplot2: Elegant Graphics for Data Analysis. Journal of the Royal Statistical Society Series A. 2011;174:245-6. doi: 10.2307/23013414.
